# Impact of routine vaccination against *Haemophilus influenzae* type b in The Gambia: 20 years after its introduction

**DOI:** 10.7189/jogh.10.010416

**Published:** 2020-06

**Authors:** Syed MA Zaman, Stephen RC Howie, Magnus Ochoge, Ousman Secka, Alasana Bah, Ignatius Baldeh, Bakary Sanneh, Saffiatou Darboe, Buntung Ceesay, Haddy Bah Camara, Fatme Mawas, Malick Ndiaye, Ilias Hossain, Rasheed Salaudeen, Kalifa Bojang, Samba Ceesay, Dawda Sowe, M Jahangir Hossain, Kim Mulholland, Brenda A Kwambana-Adams, Catherine Okoi, Siaka Badjie, Lamin Ceesay, Jason M Mwenda, Adam L Cohen, Mary Agocs, Richard Mihigo, Christian Bottomley, Martin Antonio, Grant A Mackenzie

**Affiliations:** 1 Medical Research Council Unit The Gambia at the London School of Hygiene & Tropical Medicine, Fajara, The Gambia; 2 Department of Infectious Disease Epidemiology, London School of Hygiene & Tropical Medicine, London, UK; 3 Education Department, Liverpool School of Tropical Medicine, Liverpool, UK; 4 Department of Paediatrics, University of Auckland, Auckland, New Zealand; 5 National Public Health Laboratory, Ministry of Health & Social Welfare, Kotu, The Gambia; 6 Edward Francis Small Teaching Hospital, Ministry of Health & Social Welfare, Banjul, The Gambia; 7 National Institute for Biological Standards and Control (NIBSC), Hertfordshire, UK; 8 Directorate of Health Services, Ministry of Health & Social Welfare, Banjul, The Gambia; 9 Murdoch Children’s Research Institute, Melbourne, Australia; 10 Expanded Programme on Immunization, Ministry of Health & Social Welfare, Kotu, The Gambia; 11 World Health Organization, Regional Office for Africa, Brazzaville, Republic of Congo; 12 World Health Organization, Headquarters, Geneva, Switzerland; 13 American Red Cross, Washington, D.C., USA; 14 MRC Tropical Epidemiology Group, London School of Hygiene & Tropical Medicine, London, UK; 15 Dept. of Pathogen Molecular Biology, London School of Hygiene & Tropical Medicine, London, UK; 16 Microbiology and Infection Unit, Warwick Medical School, University of Warwick, Coventry, UK; 17 Department of Disease Control, London School of Hygiene & Tropical Medicine, London, UK; 18 Department of Paediatrics, University of Melbourne, Melbourne, Australia; 19 Institut de Recherche en Sante, de Surveillance Epidemiologique et de Formation, Dakar, Senegal

## Abstract

**Background:**

In 1997, The Gambia introduced three primary doses of *Haemophilus influenzae* type b (Hib) conjugate vaccine without a booster in its infant immunisation programme along with establishment of a population-based surveillance on Hib meningitis in the West Coast Region (WCR). This surveillance was stopped in 2002 with reported elimination of Hib disease. This was re-established in 2008 but stopped again in 2010. We aimed to re-establish the surveillance in WCR and to continue surveillance in Basse Health and Demographic Surveillance System (BHDSS) in the east of the country to assess any shifts in the epidemiology of Hib disease in The Gambia.

**Methods:**

In WCR, population-based surveillance for Hib meningitis was re-established in children aged under-10 years from 24 December 2014 to 31 March 2017, using conventional microbiology and Real Time Polymerase Chain Reaction (RT-PCR). In BHDSS, population-based surveillance for Hib disease was conducted in children aged 2-59 months from 12 May 2008 to 31 December 2017 using conventional microbiology only. Hib carriage survey was carried out in pre-school and school children from July 2015 to November 2016.

**Results:**

In WCR, five Hib meningitis cases were detected using conventional microbiology while another 14 were detected by RT-PCR. Of the 19 cases, two (11%) were too young to be protected by vaccination while seven (37%) were unvaccinated. Using conventional microbiology, the incidence of Hib meningitis per 100 000-child-year (CY) in children aged 1-59 months was 0.7 in 2015 (95% confidence interval (CI) = 0.0-3.7) and 2.7 (95% CI = 0.7-7.0) in 2016. In BHDSS, 25 Hib cases were reported. Nine (36%) were too young to be protected by vaccination and five (20%) were under-vaccinated for age. Disease incidence peaked in 2012-2013 at 15 per 100 000 CY and fell to 5-8 per 100 000 CY over the subsequent four years. The prevalence of Hib carriage was 0.12% in WCR and 0.38% in BHDSS.

**Conclusions:**

After 20 years of using three primary doses of Hib vaccine without a booster Hib transmission continues in The Gambia, albeit at low rates. Improved coverage and timeliness of vaccination are of high priority for Hib disease in settings like Gambia, and there are currently no clear indications of a need for a booster dose.

Following the introduction of *Haemophilus influenzae* type b (Hib) conjugate vaccine (HCV) in many countries, there has been a substantial decline in deaths due to Hib disease globally (approximately 90%) with an estimated 299 000 deaths in 2000 and 29 500 in 2015 [1]. In 1997, The Gambia became the first African country to introduce HCV (Hib polysaccharide-tetanus toxoid conjugate vaccine) (PRP-T; Act-Hib, supplied by Pasteur Mérieux, Lyon, France) in its Expanded Programme on Immunisation (EPI) with three primary doses scheduled at 2, 3, and 4 months of age [2]. Although the specific HCV has changed over time, there has not been changes in the programme of administering three primary doses without a booster. The programme had substantial impact with no Hib disease recorded in 2002 with a dramatic decline in Hib carriage from 12% in 1997 to 0.25% in 2002 [2]. However, ongoing low incidence of the disease was reported in the West Coast Region (WCR) of The Gambia from July 2005 to April 2006 [3] and from 2008 to 2010 [4]. A resurgence of the disease was then noted in 2012-2013 as part of formal surveillance in the Basse Health and Demographic Surveillance System (BHDSS) in the rural east of the country [5]. In an earlier study among fully vaccinated children in The Gambia, Hib antibody concentrations were found to be 22% (95% CI = 4%-36%) lower in children 3 to <5 years age group compared to those in the 1 to <2 years age group [4]. Waning antibody concentrations with temporary increase in Hib disease incidence following several years of three primary doses without a booster was reported from other countries such as the UK and Mexico [6-8]. The resurgence in BHDSS, incidental disease in WCR and waning immunity in older children [4] that occurred in populations with high vaccine coverage [9] raised the question of the need for a booster dose and reinforced the need for continued surveillance [1]. Among African countries, The Gambia is well-placed to address these questions as it consistently used the same standardised methods of population-based surveillance of Hib disease and carriage for the longest period of time (periodically for over past 28 years beginning in 1990). In this study we aimed to: a. enhance Hib meningitis surveillance in WCR to obtain data comparable to historical Hib surveillance; b. continue surveillance for Hib disease in BHDSS to assess the state of disease control following the recent upsurge; c. measure the incidence of Hib disease overall and by age group; and d. undertake carriage studies in key age groups to detect potential reservoirs for increased transmission. These studies would assess any shifts in the epidemiology of Hib disease that could suggest a change in Hib vaccination policy in The Gambia or in other similar settings.

## METHODS

### Setting

**Figure 1:**
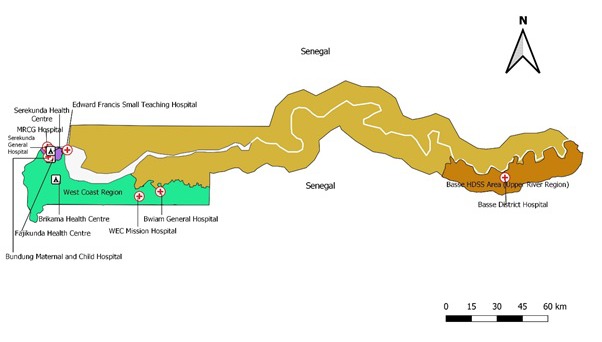
Map of Gambia showing the study sites, including the health facilities involved in *Haemophilus influenzae* type b (Hib) disease surveillance and Hib carriage studies.

The WCR on the Atlantic coast is mainly urban and peri-urban with a few small rural communities (**Figure 1**). It had a population of 168 898 aged under 5 years and 310 338 under 10 years in 2013 (Personal communication, The Gambia Bureau of Statistics, 2014). The coverages of three doses of the Hib containing pentavalent vaccine among children 12-23 months of age from 2014 to 2017 were 96%, 97%, 95%, and 92%, respectively [9].

The BHDSS was established on the southern bank of the River Gambia in 2007 (**Figure 1**) in the most eastern part of the country where the Medical Research Council Unit, The Gambia at the London School of Hygiene & Tropical Medicine (MRCG) operates a Field Station. The estimated population under 5 years of age was 34 658 in 2013 and is enumerated every four months. Basse District Hospital is the main health facility and receives referrals from five smaller health facilities in the area. There are no private inpatient facilities in the area.

### Surveillance in WCR

We carried out surveillance for Hib meningitis in WCR from 24 December 2014 to 31 March2017. The previous surveillance was carried out in the same region from May 1997 to April 2002 [2] and from October 2007 to December 2010 [4] and was limited to three hospitals: MRCG Hospital, Edward Francis Small Teaching Hospital, and World Evangelical Church Mission Hospital (**Figure 1**). Five other hospitals that began to treat suspected meningitis cases in 2012 were included in the recent surveillance: Serekunda General Hospital, Brikama Health Centre, Fajikunda Health Centre, Bundung Maternal and Child Health Hospital, and Bwiam General Hospital (**Figure 1**). We assumed that all suspected meningitis cases arising in WCR were admitted in these eight hospitals as there were no other hospitals in WCR that treated suspected meningitis cases. Vaccination status was determined from patient-held records or by the parents’ report.

Except for the addition of Real Time Polymerase Chain Reaction (RT-PCR) on cerebrospinal fluid (CSF), clinical and microbiological methods used in this surveillance were identical to previous surveillance [2,4,10]. A case of suspected meningitis was defined as a patient under-10 years of age, resident in WCR, and provisionally diagnosed as a case of meningitis by the admitting clinician. All suspected meningitis cases were admitted and managed according to national guidelines. CSF was collected for culture, Hib antigen detection by Latex Agglutination (LA), and RT-PCR. We attempted to collect blood samples for culture from all suspected meningitis cases. A case of Hib meningitis was defined as a suspected meningitis case from whom Hib was detected in CSF by culture or LA or in blood by culture. We also defined Hib meningitis plus-PCR if Hib was detected in CSF by culture, antigen detection, or RT-PCR, or in blood by culture. All laboratory assays were performed at MRCG Fajara laboratories.

*CSF culture and antigen detection by LA:* For hospitals that were within one-hour drive from the MRCG laboratories, CSF was collected without transport media. Where CSF could not be transported within an hour, it was inoculated into Trans Isolate Media [11], stored in a 37°C incubator, and transported to MRCG laboratories within 48 hours. CSF culture was done using standard methods that remained unchanged from previous surveillance. Antigen detection by LA (Directigen^TM^ Meningitis Combo Test, Beckton Dickinson, Erembodegem, Belgium) was performed on CSF supernatants following the manufacturer’s instructions.

*Blood culture:* Blood culture used an automated Bactec system (Becton Dickinson, Sparks, USA). For alarm positive blood cultures, a 50-100μl aliquot of the specimen was inoculated onto Blood Agar, Chocolate Agar and MacConkey Agar plates, streaked, and incubated at 37°C with and without 5% CO_2_ for 24-48hrs for isolation of the causative agents of bacterial meningitis [12]. All suspected Hib growth on Chocolate Agar plates were confirmed by Oxidase test, X+V growth factor requirement, and serotyped using Hib antisera (Statens Serum Institute, Denmark).

*RT-PCR:* CSF for RT-PCR was transported to the MRCG laboratories within an hour. Otherwise, aliquots were stored at -20°C until transported. DNA was extracted from CSF as described by Carvalho et al [13]. The RT-PCR assay targeted the protein D-encoding gene (*hpd*) for detection of both typeable and non-typeable *Haemophilus influenzae* [14]. All detections with a cycle threshold ≤36 were considered positive. Valid results required proper functioning of controls and excluded data flagged by the QuantStudio^TM^ 7 Flex software (Thermo Fisher Scientific version 1.2). Hib serotype was identified by screening for the presence of the target gene bcsB, as previously described [14].

### Surveillance in BHDSS

In May 2008, surveillance for invasive bacterial disease was established within BHDSS [15]. All individuals with suspected meningitis, and all BHDSS residents aged ≥2months presenting at one of the health facilities in BHDSS with suspected pneumonia, sepsis, or meningitis were investigated. Surveillance continued 24 hours a day, 7 days a week. Case ascertainment used standardised criteria at each of the steps: (1) nurse screening for referral to a clinician, (2) surveillance diagnosis by a clinician and (3) investigation [16]. In contrast to WCR, blood culture was performed for all patients with suspected pneumonia, sepsis, or meningitis and lumbar puncture was done in cases of suspected meningitis. Aspiration of pleural fluid or lung aspiration was performed for selected patients. Vaccination status was determined from patient-held records until 2011 and thereafter this was recorded in an electronic database at the immunisation clinics.

Since 2013, patients with invasive bacterial disease have been tested for HIV by government services. We used an automated Bactec system to culture blood and a small proportion of samples collected at night in outlying clinics were cultured by traditional microbiological methods [16]. Other clinical samples were processed using standard methods that remained unchanged throughout the surveillance period [17]. Identification of Hib and other encapsulated Hi was done by slide agglutination using polyvalent and monovalent antisera to Hi types a, b, c, d, e and f (Beckton Dickinson, Erembodegem, Belgium). We defined a case of Hib disease as a patient with suspected pneumonia, sepsis, or meningitis from whom Hib was isolated from a normally sterile site. All samples were processed at the MRCG Basse laboratories. Laboratories in Basse and Fajara submitted to external QA procedures through the UK National External Quality Assessment Service (Sheffield, UK).

### Carriage study

The carriage study was performed from July 2015 to November 2016. We aimed to collect oropharyngeal swabs (OPS) from 1000 children per age group (12-23 months, 2-7 years, and 8-16 years) with 500 from WCR and 500 from BHDSS. We calculated that this would provide 80% power to detect a hypothesised increase in carriage from 1% to 3% in children aged 12-23 months, and would provide precision of at least ±1% for all estimates. As there were no date of birth records for many school children, we enrolled them based on their class and grade level. For the pre-nursery children, OPS samples were collected from consecutive children attending immunisation clinics for measles vaccination at Serekunda Health Centre (SHC) in WCR and at Basse District Hospital (BDH) in BHDSS after obtaining written informed consent from primary carers. SHC and BDH are among the largest immunisation clinics located centrally in WCR and BHDSS respectively (**Figure 1**).

We used lists of nurseries and schools to enumerate children, starting with nurseries and schools located closest to SHC or BDH and moved centrifugally until approximately equal numbers of children were identified in each grade (grades 1-4 for nurseries and 1-10 for schools). Study aims and procedures for collection of OPS were explained to the teachers and then to the children. OPS were collected from children in the classrooms in presence of the teachers. Microbiological methods for the isolation and identification of Hib from OPS have been described elsewhere [4].

### Statistical analysis

Incidence rates of Hib meningitis and Hib meningitis plus-PCR in WCR were estimated for 2015 and 2016 and for the age groups 1 month-4 years and 5-9 years. Person-time for this analysis were calculated using 2013 WCR census data adjusted for estimated population growth [16].

The BHDSS analysis of incidence rates of Hib disease was limited to children 2-59 months of age who were resident in the BHDSS, and person-time was calculated using mid-year population estimates.

Carriage prevalence with exact 95% confidence intervals was calculated for age groups 12-23 months, 2-7 years and 8-22 years, and estimates were compared using Fisher exact test. In all analyses, a statistical significance level of 5% was used.

### Ethics

BHDSS and WCR surveillance, and the carriage study were approved by The Gambia Government/MRC Joint Ethics Committee (SCC/EC 1087, 1247; SCC/EC 1387; L2016/10).

## RESULTS

### Surveillance in WCR

**Figure 2:**
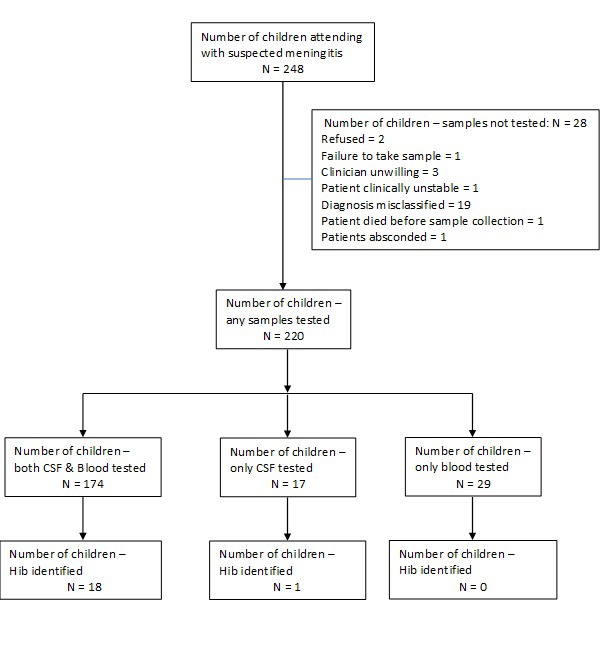
Flow of children with suspected meningitis investigated in *Haemophilus influenzae* type b disease surveillance in the West Coast Region (WCR) of The Gambia from December 2014 to March 2017.

In WCR, 248 children were admitted with suspected meningitis from 24 December 2014 to 31 March 2017 (**Figure 2**). The median age of suspected meningitis cases was 2.8 years (inter quartile range (IQR) = 10.0 months-6.0 years).

Using conventional microbiology, five Hib meningitis cases were detected. An additional 14 cases were detected by RT-PCR alone (**Table 1**). The median age differed among the cases detected by conventional microbiology (10.5 months, IQR = 10.1-10.8) and by RT-PCR alone (60.0 months, IQR = 14.5-81.0). Of 19 cases with Hib meningitis plus-PCR, two (10%) were too young to be protected by vaccination. Of 17 cases old enough to be protected by vaccination, seven (41%) were unvaccinated and two (12%) were under-vaccinated for their age. Of the seven unvaccinated cases, five were aged ≥5 years. Of the eight fully vaccinated cases, three were aged <2 years, three between two and four years, and two ≥5 years.

**Table 1:** Characteristics of *Haemophilus influenzae* type b (Hib) meningitis cases in the West Coast Region (WCR) of The Gambia, admitted in health facilities from December 2014 to March 2017*

Admission year	Admission month	Age (months)	Gender	Number of Hib vaccine doses	Age (months), 1^st^ dose†	Age (months), 2^nd^ dose†	Age (months, 3^rd^ dose†	Samples obtained	Positive assays	Outcome of hospitalisation
2014	Dec	59	Male	3	2	3	4	CSF & Blood	RT-PCR	Recovered
2015	Jan	77	Male	3	3	5	6	CSF & Blood	RT-PCR	Recovered
2015	Feb	60	Male	0				CSF & Blood	RT-PCR	Died
2015	Mar	60	Male	0				CSF & Blood	RT-PCR	Died
2015	Mar	82	Male	3	2	3	5	CSF & Blood	RT-PCR	Recovered
2015	Mar	89	Female	0				CSF & Blood	RT-PCR	Recovered
2015	Mar	2	Female	1	2			CSF & Blood	RT-PCR	Recovered
2015	Apr	14	Male	3	10	12	12	CSF & Blood	RT-PCR	Recovered
2015	Apr	3	Male	2	2	3		CSF & Blood	RT-PCR	Recovered
2015	Apr	40	Male	3	2	3	4	CSF & Blood	RT-PCR	Recovered
2015	Jul	10	Female	1	2			CSF & Blood	Blood & CSF culture, Latex, RT-PCR	Recovered
2015	Jul	10	Male	3	2	3	4	CSF & Blood	RT-PCR	Recovered
2015	Jul	81	Female	0				CSF & Blood	RT-PCR	Recovered
2015	Aug	90	Female	1			53	CSF & Blood	RT-PCR	Recovered
2016	Mar	10	Female	0				CSF & Blood	Latex & RT-PCR	Recovered
2016	Apr	60	Male	0				CSF & Blood	RT-PCR	Recovered
2016	Aug	26	Male	3	2	3	4	CSF & Blood	Blood culture	Died
2016	Sep	10	Male	0				CSF & Blood	Latex	Recovered
2016	Sep	8	Male	3	2	3	4	CSF only	Latex	Recovered

RT-PCR – real time polymerase chain reaction, CSF – cerebrospinal fluid, Latex – latex agglutination

*Hib was detected in CSF by culture, antigen detection by Latex, or RT-PCR, or in blood by culture. No Hib meningitis cases were detected from January to March 2017.

†Dose of Hib vaccine.

Three cases of Hib pneumonia were reported incidentally from the MRCG hospital where blood culture is normally done for cases of suspected invasive bacterial disease. These cases were not included in any analysis.

**Figure 3:**
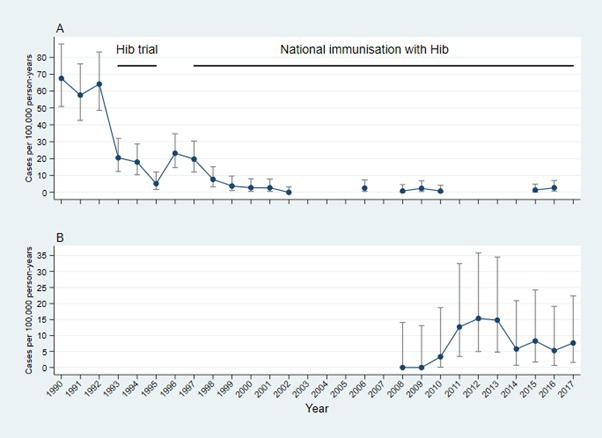
Incidence of *Haemophilus influenzae* type b (Hib). **Panel A.** Incidence of *Haemophilus influenzae* type b (Hib) meningitis in children under 5 years of age in the West Coast Region using conventional microbiology, 1990-2016. **Panel B.** Incidence of Hib disease in children aged 2-59 months in the Basse Heath and Demographic Surveillance System, 2008-2017.

The incidence of Hib meningitis in those aged 1-59 months was 0.7 and 2.7 per 100 000 CY in 2015 and 2016 respectively, similar to the incidence of 1.3 per 100 000 child years (CY) between 2008 and 2010 as previously reported [4](**Figure 3**, Panel A). Incidence was higher in under-5s compared to older children. The median age of cases was 10.4 months (IQR = 10.1-10.8).

The incidence of Hib meningitis plus-PCR in 2015, among children aged 1 month-9 years was 4.1 per 100 000 CY (**Table 2**). Incidence was higher in the under-5s compared to older children (*P* = 0.33). In 2016, the incidence was 1.6 per 100 000 CY. As in 2015, incidence was higher in under-5s compared to older children (*P* = 0.02).

**Table 2:** Incidence rates of *Haemophilus influenzae* type b (Hib) meningitis in the West Coast Region (WCR) of The Gambia by laboratory methods of diagnosis, calendar year, and age groups in 2015 and 2016

Year	Age	Child-years	Cases*	Rate (95% CI)†
**Conventional microbiology**‡ **or RT-PCR of CSF:**				
2015	1m-4yrs	148 808	8	5.4 (2.3,10.6)
	5-9yrs	165 114	5	3.0 (1.0,7.1)
	1m-9yrs	313 922	13	4.1 (2.2,7.1)
2016	1m-4yrs	146 159	5	3.4 (1.1,8.0)
	5-9yrs	165 317	0	0.0 (0.0,2.2)
	1m-9yrs	311 476	5	1.6 (0.5,3.7)
**Conventional microbiology only:**				
2015	1m-4yrs	148 808	1	0.7 (0.0,3.7)
	5-9yrs	165 114	0	0.0 (0.0,2.2)
	1m-9yrs	313 922	1	0.3 (0.0,1.8)
2016	1m-4yrs	146 159	4	2.7 (0.7,7.0)
	5-9yrs	165 317	0	0.0 (0.0,2.2)
	1m-9yrs	311 476	4	1.3 (0.3,3.3)

RT-PCR – real time polymerase chain reaction, CI – confidence interval, CSF – cerebrospinal fluid

*Hib meningitis cases. Excluding one case detected in December 2014.

†Rate per 100 000 child-years.

‡Conventional microbiology: Hib was detected by culture of cerebrospinal fluid (CSF) or blood, or by antigen detection by latex agglutination of CSF.

### Surveillance in BHDSS

Surveillance enrolled 22 262 children who met criteria for referral and were assessed by clinicians. Of those children, 21 552 had a surveillance diagnosis of suspected pneumonia, sepsis, or meningitis and 21 315 had specimens collected for microbiological analysis. Twenty-five cases of Hib disease were detected from 2008 to 2017 (**Table 3**). Their median age was five months (IQR = 3-15). Nine children (28%) were too young to be protected by vaccination. Of 16 children old enough to be protected by vaccination, five were under-vaccinated for their age, four with vaccine failure after three doses and five with vaccine failure after two doses. Of the four fully vaccinated children, three were aged <2 years and one was between two and four years of age. HIV was tested in eight cases or their mothers, and two mothers were found to be positive. Incidence of Hib disease peaked in 2012 and 2013 at 15.0 per 100 000 CY (**Figure 3**, Panel B) but fell in 2014 and remained stable between 5.0 and 8.0 per 100 000 CY from 2014 to 2017.

**Table 3:** Characteristics of children aged 2-59 months with *Haemophilus influenzae* type b (Hib) disease resident in the Basse Health and Demographic Surveillance System (BHDSS), Upper River Region, The Gambia, admitted in health facilities from 2008 to 2017

Admission year		Admission month	Age (months)	Gender	Number of Hib vaccine doses	Age (months), 1^st^ dose*	Age (months), 2^nd^ dose*	Age (months), 3^rd^ dose*	Positive samples	Outcome of hospitalisation	HIV test
2010	Jul		29	Male	0				Blood	Recovered	ND
2011	Jun		3	Male	1	3			CSF & Blood	Died	ND
2011	Aug		2	Female	0				CSF & Blood	Recovered	ND
2011	Apr		15	Male	3	1	3	5	CSF & Blood	Recovered	ND
2011	Oct		11	Female	3	2	4	6	LA	Recovered	ND
2012	Jul		22	Female	3	1	5	5	Blood	Died	ND
2012	Sep		54	Female	0				Blood	Not admitted	ND
2012	Oct		4	Female	2	2	4		CSF & Blood	Discharged	ND
2012	Nov		48	Male	0				Blood	Discharged	ND
2012	Jun		5	Female	2	NA			Blood	Recovered	ND
2013	Jan		48	Female	0				CSF & Blood	Discharged	ND
2013	Feb		3	Female	1	2			Blood	Discharged	Mo
2013	Apr		2	Female	0				Blood	Discharged	ND
2013	May		2	Female	0				Blood	Discharged	ND
2013	Oct		14	Female	0				Blood	Died	Mo
2014	Feb		2	Female	0				Blood	Discharged	ND
2014	Sep		3	Female	1	2			Blood	Discharged	Mo
2015	Apr		5	Female	2	3	5		Blood	Not admitted	Mo
2015	Jul		6	Male	2	4	6		Blood	Discharged	ND
2015	Aug		38	Male	3	2	3	4	Blood	Discharged	Inf
2016	Mar		4	Male	1	3			Blood	Died	Mo
2016	Sep		2	Male	1	2			Blood	Discharged	Mo
2017	Jan		11	Female	1	NA			Blood	Recovered	ND
2017	Jun		3	Female	1	NA			CSF & Blood	Recovered	ND
2017	Oct		4	Female	2	3	4		Blood	Recovered	Mo

NA – not available, CSF – cerebrospinal fluid, LA – lung aspirate, ND – not done, Mo – mother’s HIV test result, Inf – infant’s HIV test result

*Dose of Hib vaccine.

### Hib Carriage

OPS were collected from 3157 children with a median age of 72.0 months (IQR = 20.5-121.8). Hib was isolated from eight children: one aged 1.2 years, one 4.5 years, and six between 8.1 and 17.1 years (**Table 4**). Six (75%) of the eight children were fully vaccinated. Overall prevalence of carriage was 0.12% in WCR and 0.38% in BHDSS. Carriage increased significantly with age in BHDSS (*P* = 0.05) but not in WCR (*P* = 0.55).

**Table 4:** Prevalence of *Haemophilus influenzae* type b (Hib) carriage in 2015/2016 by region of The Gambia and age groups

Region	Age groups	Gender (% male)	Number swabbed	Number with Hib carriage	Prevalence of carriage (%) (95% Cl)
WCR	12-<24 months	49	503	1	0.20 (0.005-1.1)
	2-7 years	51	537	0	0 (0-0.7) *
	≥8 years	43	532	1	0.19 (0.005-1.0)
BHDSS	12-<24 months	52	491	0	0 (0-0.75) *
	2-7 years	48	542	1	0.18 (0.005-1.0)
	≥8 years	48	552	5	0.91 (0.30-2.1)

CI – confidence interval, WCR – West Coast Region, BHDSS – Basse Health and Demographic Surveillance System

*One-sided, 97.5% confidence interval

## DISCUSSION

We report the incidence of Hib meningitis over a 28-year period in WCR of The Gambia, the last 20 years of which saw the introduction of three primary doses of HCV without a booster into the EPI. Incidence of Hib meningitis in under-5s in WCR remained low at 0.7 and 2.7 per 100 000 CY for 2015 and 2016 respectively, similar to incidence estimated in the same area using similar methods from 2008 to 2010 [3] and in 2001 and 2002 [2] (**Figure 3**, panel A). The results of this study substantiate long-term data from other African countries, which show that three primary doses of HCV without a booster have caused substantial and sustained declines in disease incidence. However, this study also adds data to other long-term population-based surveillance studies [18], which show that current vaccination strategies with three primary doses without a booster may not eliminate Hib transmission. Following the resurgence of Hib disease in BHDSS in 2012 and 2013, disease incidence fell to a stable level between 5.0 and 8.0 per 100 000 CY (**Figure 3**, Panel B). Carriage prevalence remains low at 0.2% in 12-23 months age group in WCR which is similar to previously reported estimates of 0.9% in 2009 [4] and 0.3% in 2002 [2].

Incidence of Hib meningitis in WCR was higher when we included additional Hib meningitis cases identified by RT-PCR (5.4 and 3.4 per 100 000 CY for 2015 and 2016 respectively) (**Table 2**). Of a total of 22 cases only eight were detected without the use of PCR and PCR yielded 14 additional cases. PCR is a sensitive and specific diagnostic tool that may be valuable in populations with high pre-hospital antibiotic use. In a recent study of 411 children presenting with suspected sepsis or severe focal infections in the largest referral hospital in The Gambia, 80% reported prehospital antibiotic use [19]. Indeed, the use of PCR should be considered to increase the sensitivity of any surveillance for Hib disease.

Our findings of Hib meningitis incidence of 4.0-5.0 per 100 000 CY in WCR and Hib disease incidence of 5.0-8.0 per 100 000 CY in BHDSS represent greater than 90% reductions from pre-vaccine Hib meningitis incidence of 60.0-70.0 per 100 000 CY (**Figure 3**). Similar vaccine impact in settings using a three dose primary schedule without a booster have been described in Kenya [18], Chile, Colombia [20], and Mongolia [21]. In all these settings the estimated incidence of Hib disease in the under-5-year age group three or more years after vaccine introduction was <2.0 per 100 000 CY. Hib disease is uncommon in these settings because of the effectiveness of Hib vaccination with three primary doses without a booster.

Despite the undoubted impact of Hib vaccination in our setting, detection of ongoing Hib cases and reports of Hib mortality globally [1] are concerning. More rapid waning of immunity associated with the use of three primary doses without a booster is one reason to consider the use of booster doses. Our data, however, indicate that the disease in The Gambia is associated with other factors that may be amenable to public health intervention. Of the WCR cases over two years of age, five were unvaccinated, five were fully vaccinated, and one received only one dose, indicating that waning immunity and unvaccinated status were both similarly associated with disease. In BHDSS, only one case occurred in fully vaccinated cases older than two years of age.

Rather than waning immunity, Hib cases were also related to issues of coverage and timeliness. Thirty-seven percent of all the cases in WCR (n = 19) and 36% in BHDSS occurred in unvaccinated children. These children were Gambian residents at the time of illness, although some had migrated to The Gambia and were not vaccinated in their country of birth. Our data indicate that indirect protection offered by high coverage of three primary doses does not completely protect these unvaccinated children. Children who do not present for vaccination, and those who migrate after 12 months of age, represent a challenge to the EPI. Strategies to identify and give missed doses at visits when routine vaccination is not given could be considered. Similarly, promoting the value of vaccination and increasing access to care may increase coverage in households that are difficult to reach. Addressing the timeliness of vaccination is challenging in our setting where most vaccinations are delivered at mobile clinics that visit small geographic catchment areas on a monthly basis.

Delayed vaccination was associated with a greater number of cases than being unvaccinated. Of 17 cases in WCR, and 16 in BHDSS who were old enough to be protected by the 2, 3, 4-month schedule, the first or second doses were delayed for two in WCR and eight in BHDSS. In WCR, two cases, at 2.7 and 3.7 months of age, occurred before the age at which children could be protected by the current schedule. In BHDSS nine of 25 cases were too young to be protected by vaccination and five were aged 3 or 4 months. To prevent Hib cases at 3 or 4 months of age, the EPI could transition to scheduling at 6, 10, and 14 weeks of age as in neighbouring Senegal [22], or even 1, 2, and 3 months of age as in Papua New Guinea [22] to maximise individual level protection, but there is no empirical data to assess the added benefit of early primary dosing at population level.

A notable finding of this study was that the median age of Hib cases in WCR was 3.4 years, which is in contrast to the median age of <1 year seen both in previous WCR surveillance reports [4,10] and the BHDSS. Hib disease in older children in a population with low carriage is difficult to explain. We may be observing a shift in the epidemiology of Hib disease in The Gambia, and this must be closely watched. It is also possible that there were upward shifts in the age distribution of cases in the previous surveillance in WCR (2008-2010) and BHDSS, but insensitive conventional microbiology tests were unable to capture all cases, the majority of which might have been >1 year of age.

It is not clear why the resurgence of the Hib disease occurred in BHDSS. Potential explanations include increased acquisition of Hib by older children due to their waning immunity and subsequent transmission to younger unvaccinated/partially vaccinated children, temporal changes in the circulation of respiratory viruses associated with bacterial pneumonia, or cross border migration of poorly vaccinated populations. It may also be that the increase in Hib cases in BHDSS was a chance finding associated with small numbers of cases and unstable year-to-year estimates. It does not appear that changes in the prevalence of HIV infection had a role. The BHDSS surveillance, which does include older children and adults, detected only two older cases, one in a 10-year-old in 2012 and the other in a 5-year-old in 2013. Low susceptibility and less intense interaction (due to poor schooling rates) among older children in the rural setting may explain low Hib disease rates.

The prevalence of carriage (carriage) was very low in younger age groups and was similar to previous surveillance. However, it was not clear why carriage was relatively higher (0.91%) in older children in BHDSS. Nonetheless, carriage <1% among older children does not indicate a specific age group responsible for Hib transmission in the population. Low carriage among older children is consistent with observations elsewhere [18] indicating the schedule of three primary doses without a booster can exert substantial indirect effects. An earlier Gambian study showed significantly higher concentrations of Hib antibodies in children in urban areas compared to those living in rural areas, and antibody concentrations were significantly lower in older compared to younger children [4]. These two factors could explain the relatively higher carriage in older children in the rural BHDSS. HIV infection is unlikely to be driving Hib carriage in The Gambia since HIV prevalence is very low. Relatively higher carriage observed in older children in the BHDSS might have served as a reservoir of Hib infection for transmission to younger children resulting in the resurgence of invasive Hib disease in 2012 and 2013. However, the overall low carriage in BHDSS is evidence that the current schedule is associated with good herd immunity.

This study had some limitations. We could not include Hib cases with pneumonia or septicaemia in WCR because of inadequate resources in the hospitals. This limitation has been consistent across the surveillance periods evaluating the impact of the introduction of Hib vaccination in WCR. However, it is also true that surveillance for Hib pneumonia is difficult due to challenges in implementing a standardised definition for pneumonia and due to low yield of blood cultures from patients with pneumonia. Surveillance for Hib meningitis is somewhat more efficient because CSF from patients with suspected meningitis will be positive more often than blood cultures from patients with pneumonia. Thus, use of meningitis as an endpoint is less sensitive to ascertainment bias. Another limitation was not selecting children for the carriage study as a random sample of the population. Nevertheless, these children came from the same population from which the cases arose, and school-based sampling is an accepted survey method [23]. We could not exclude the possibility of the emergence of a hypervirulent Hib strain that might have caused Hib disease since we have not genotyped all the isolates. However, any hypervirulent stains were not detected in an earlier study investigating the re-emergence of Hib disease following its elimination [3].

A strength of the study was its ability to estimate incidence in WCR and BHDSS. A further strength of the study was the use of consistent surveillance methods in WCR allowing comparison with earlier surveillance in 2001 and 2002, and from 2008 to 2010, and continuous observation in the BHDSS from 2008 to 2017.

Taken together, these data suggest that three primary doses of Hib vaccine in infants without a booster is effective in sustaining the control of Hib disease in The Gambia. However, the sustained low incidence of disease in this population is a concern for the risk of a future upsurge and for the failure to eliminate the disease. Experience from the UK suggests that there are settings where a booster dose is needed to sustain reductions in Hib disease. However, our data were insufficient to indicate the use of a booster dose. There is little experience of a booster dose in resource poor settings such as The Gambia. South Africa introduced a booster dose in 2010, 11 years after introduction of three primary series of Hib vaccine, but this was not in response to low Hib antibody titres but rather due to the introduction of a combination vaccine including inactivated polio vaccine [24]. Before the introduction of the booster in South Africa, an increase in Hib disease incidence was noted over several years, although this increase was small and disease incidence remained within the range reported from other countries.

Interventions to further control of Hib disease in our setting would aim to improve coverage – catch-up missed doses, engage hard to reach households and migrants, and improve the timeliness of the first and second doses of Hib vaccine. In addition, continued surveillance is needed to monitor whether effective control of disease is sustained or whether shifts in epidemiology (eg, disease occurring in older children and fewer opportunities for natural boosting) will necessitate a booster dose. Information about Hib disease epidemiology in The Gambia with long term use of HCV can inform recommendations for Hib vaccination schedules in similar settings. In the UK, an increase in vaccine failure 8 years after the introduction of routine vaccination was at least partly caused by a greater than expected decline in Hib antibody response following a schedule without a booster dose. Therefore, we also need to undertake studies periodically to confirm long-term immunity by monitoring Hib antibody response and carriage.
